# One-stage technique for sagittal split ramus osteotomy combined with mandibular angle ostectomy

**DOI:** 10.1038/s41598-018-19141-3

**Published:** 2018-01-29

**Authors:** Shuo Chen, Yi Zhang, Jin-gang An, Yang He

**Affiliations:** Department of Oral and Maxillofacial Surgery, Peking University School and Hospital of Stomatology; National Engineering Laboratory for Digital and Material Technology of Stomatology, Beijing Key Laboratory of digital Stomatology, Beijing, 100081 China

## Abstract

Bilateral sagittal split ramus osteotomy (BSSRO) is commonly used to correct mandibular prognathism or retrognathism. Patients with mandibular prognathism or retrognathism may also present with a prominent mandibular angle. In this paper, we share our experience on BSSRO with mandibular angle resection. Eleven patients who were treated from July 2014 to December 2016 were included in this study. The mandibular angle was resected through the medial side of the mandible after BSSRO. The mandibular angle measurements of the patients changed significantly after surgery (p < 0.05). Unanticipated fractures and mandibular hematoma did not occur. Therefore, BSSRO combined with mandibular angle ostectomy through the medial side of the mandible can be used to safely and effectively correct facial deformity.

## Introduction

Bilateral sagittal split ramus osteotomy (BSSRO) is commonly used to correct mandibular deformities, such as mandibular prognathism or retrognathism. Patients diagnosed with mandibular prognathism or retrognathism may also present with a prominent mandibular angle. Resecting the mandibular angle requires subperiosteal dissection from the posterior to the inferior margin of the mandible to expose the proposed osteotomy site over the lateral mandibular ramus and angle^1,2^. If mandibular angle ostectomy is performed simultaneously with BSSRO, stripping the mucoperiosteum and pterygomasseteric sling may increase the risk of intraosseous ischemia and the necrosis of proximal segment^3,4^.

In this article, we share our experience in the simultaneous performance of BSSRO with mandibular angle resection through the medial side of the mandible.

## Results

The random errors for the angular measurements ranged from 0.37° to 0.94°. The paired t-test between two times showed no significant difference at p = 0.05.

Eleven patients were included in this study. Their general information is presented in Table 1. The mean follow-up period of the patients was 10 months (from 6–13 months). All patients were satisfied with the outcome of the procedure. Their wounds healed uneventfully, except in one patient who suffered from mucosal dehiscence at one side of the mandibular body. This complication was conservatively managed by the application of pressure and immobilization of the area. In five patients, the inferior alveolar nerve (IAN) was exposed during operation (eight sides) without transection in any case. Unanticipated fractures and mandibular hematoma did not occur.

**Table 1:** General patient information.

Study Variables	Values
Sample size	11
Gender, n (%)	
Male	4
Female	7
Age (year), mean ± SD (range)	23.3 ± 2.0; (21–27)
BSSRO	11
Le Fort I osteotomy	9
Genioplasty	11
Bilateral mandibular angle ostectomy	11

**Figure 1:**
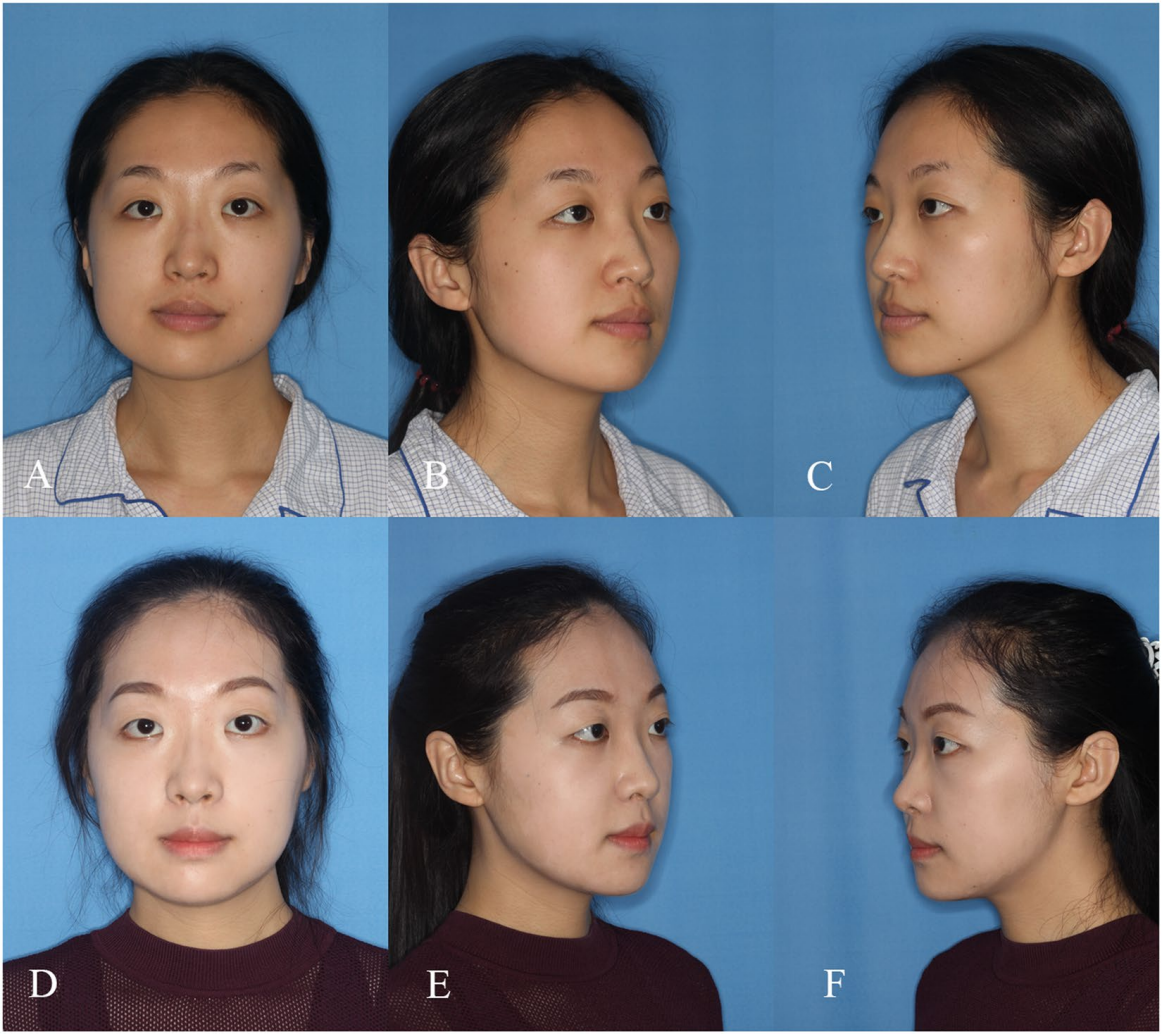
Patient with facial asymmetry deformity. Le Fort I osteotomy and BSSRO were performed to correct the cant of the occlusal plane. Unequal amounts of bone from each side were removed by resecting the mandibular angle through the medial side of the mandible. (**A**) Frontal view of the preoperative photograph. (**B**) and (**C**) Oblique view of the preoperative photograph. (**D**) Frontal view of the 6-month postoperative photograph. (**E**) and (**F**) Oblique view of the 6-month postoperative photograph.

The mandibular angle changed significantly with time (p < 0.05) (Table 2, Fig. 1). The measurements increased from T0 (before surgery) to T1 (immediately after surgery) and indicated that the mandibular angle changed during surgery. Meanwhile, the difference between T1 and T2 (3 months after surgery) signified that the mandibular angle increased significantly. However, the measurements showed no significant difference between T2 and T3 (10 months on average after surgery).

**Table 2:** Mandibular angle measurements at different stages (n = 11).

	T0	T1	T2	T3
Mandibular angle (°)	107.4 ± 2.9^a^	120.8 ± 1.8^b^	124.3 ± 2.4^c^	124.7 ± 2.4^c^

^a,b,c^Identical superscripts indicate no significant difference among the indicated groups (p > 0.05). Adjustment for pair-wise multiple comparisons was applied through the Bonferroni test.

## Discussion

In this study, we evaluated the treatment outcomes of patients who underwent BSSRO combined with mandibular angle ostectomy. The mandibular angle increased significantly after surgery. All the patients were satisfied with the outcome, and no additional complications were observed.

BSSRO was first described by Trauner and Obwegeser^5^, modified by Dal Pont^6^, and further refined by Hunsuck^7^ in 1968 and Epker^8^ in 1977. Horizontal osteotomy is first performed through the cortical bone and superior lingula and then extended to the posterior border of the ascending ramus following the method described by Obwegeser and Dal Pont. Given that the internal osseous reinforcement is considerably weakened in the region immediately posterior to the mandibular foramen, Hunsuck terminated the horizontal osteotomy posterior to the lingula. This modification typically splits the mandible posterior along the inferior alveolar foramen. However, even with the most careful precautions, imperfect bone division is not uncommon^9^.

To guarantee that the mandibular angle is intact with the proximal segment, we performed an additional osteotomy vertical to the horizontal osteotomy and modified the splitting procedure. We used a slightly curved chisel to straddle the horizontal osteotomy and performed the initial split at the posterior aspect of the foramen, i.e., we split the posterior border of the mandible along the mylohyoid groove. This procedure is different from the conventional method, in which the osteotome progresses from the anterior to the posterior positions to complete the split. Typically, the anterior ridge of the ramus should be reduced with a large trimming bur before osteotomy to acquire a good overview of the lingual side of the mandible.

After splitting, the angle can be removed through the medial side of the mandible. This technique minimally detaches the mucoperiosteum and pterygomasseteric sling from the proximal segment and avoids impaired blood flow and resultant sequelae. During the procedure, the surgical site must be completely exposed by the surgical assistants. A lateral bending hook is usually placed between the proximal and distal segments at the posterior border of the ramus to enlarge the gap between segments and protect the inferior alveolar neurovascular bundle. In this procedure, the incidence of IAN exposure was 36.4% (8/22), which was within the range of 18.4% to 70% reported in the literature^10,11^. Moreover, the IAN was not transected. Therefore, professional and careful operation would not increase the risk of IAN injury during mandibular angle osteotomy. Hematoma occasionally occurs during mandibular angle resection^1^. Subperiosteal manipulation would protect soft tissue from injury, and no substantial bleeding would occur. Surgical skill and experience remain important when the reciprocating saw is used to finish the ostectomy of the mandibular angle.

The mandibular angle measurements increased after surgery, thus making the square-shaped mandibular angle appear rounded with a curved contour. The increased mandibular angles between T1 and T2 may be related to bone remodeling and resorption at the margins of the osteotomy lines. The remodeling of bone in adult humans is realized in a regulated interplay between osteoblasts and osteoclasts. The total time span of a bone remodeling cycle is lasted 90 to 120 days^12^. Therefore, the measurements showed no significant difference between T2 and T3, suggesting that after the surgery, the bone condition of mandibular angle tended to be stable three months later.

Bone grafting is often required during Le Fort I osteotomy to 1) fill the gap between segments and improve stability after maxillary advancement^13^ and 2) increase the volume of the paranasal space and improve postoperative aesthetic results^14^. The resected bone from the medial aspect of the mandibular angle in the proximal segment can provide local autogenous bone for grafting. Bone from the medial side of the proximal segment can be obtained without changing the mandibular angular profile when monocortical bone is removed from the mandibular angle. Therefore, depending on the situation, the surgeon can remove bicortical or monocortical bone from the mandibular angle after splitting.

In conclusion, BSSRO combined with mandibular angle ostectomy through the medial side of the mandible can be used to safely and effectively correct facial deformity.

## Patients and Methods

### Subjects

All subjects underwent orthognathic surgery at our hospital over the period of July 1, 2014 to December 31, 2016. Informed consent to publish identifying information/images was obtained from each subject. The study was approved by the Institutional Review Board of Peking University School and Hospital of Stomatology. We confirm that all methods were performed in accordance with the relevant guidelines and regulations. Orthognathic surgery procedures included BSSRO with or without Le Fort I osteotomy or genioplasty. The inclusion criteria covered patients receiving BSSRO combined with mandibular angle ostectomy. Patients with deformity secondary to trauma, cleft lip and palate, or systemic disease were excluded.

### Surgical technique

**Figure 2:**
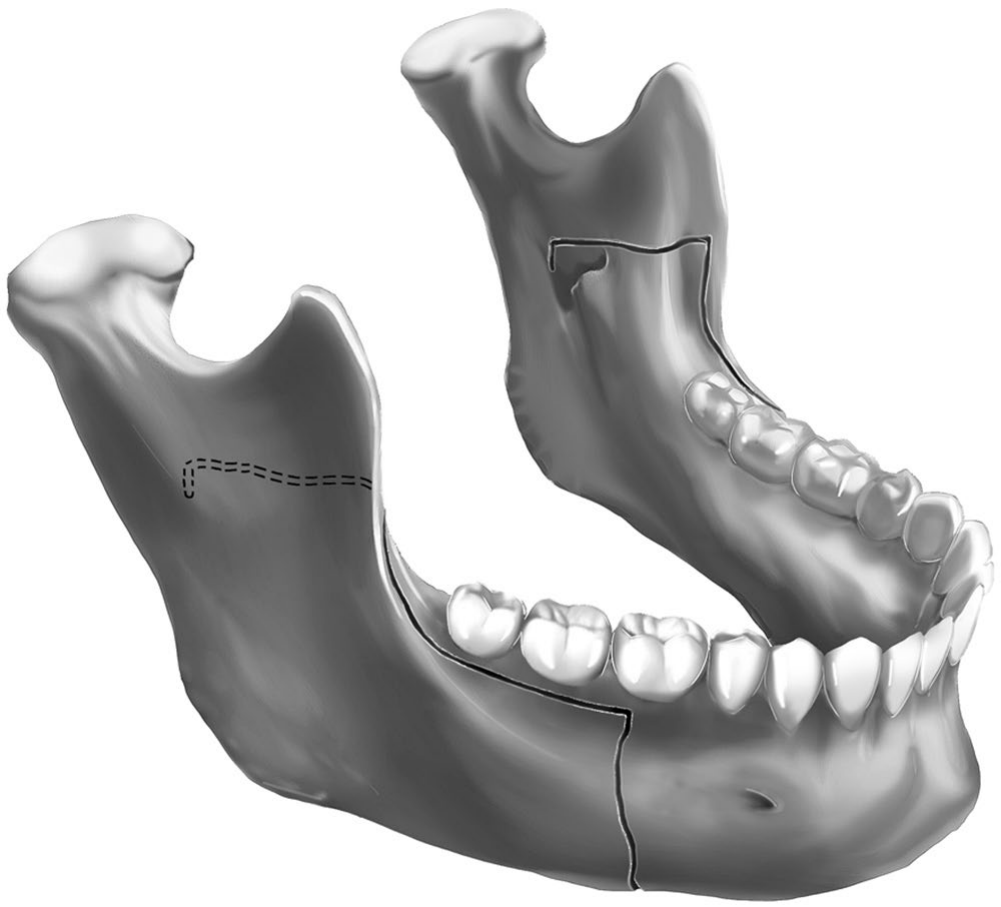
Additional osteotomy vertical to the horizontal osteotomy.

**Figure 3:**
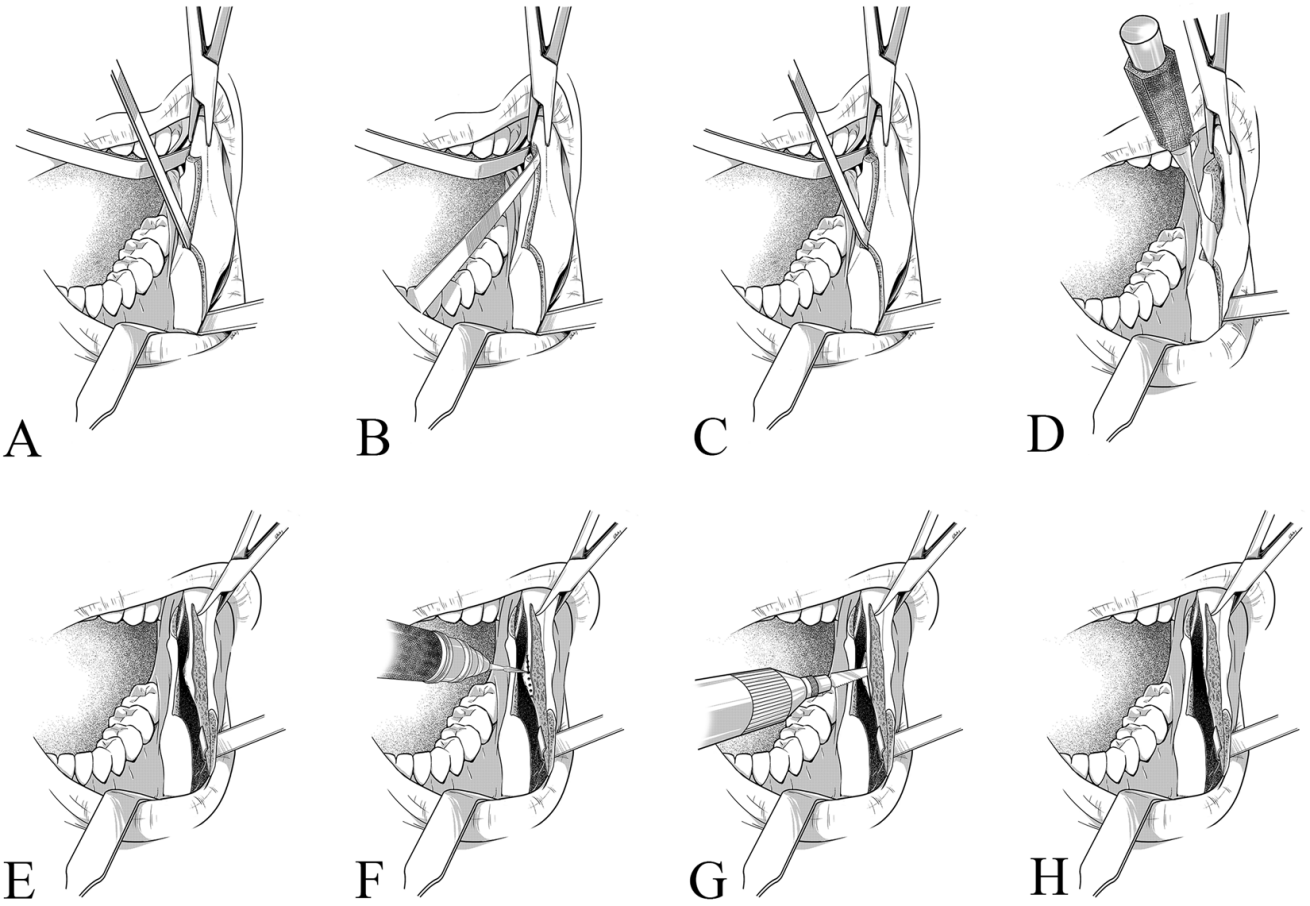
Procedure for sagittal splitting and mandibular angle ostectomy. (**A**) A straight chisel defines the osteotomy cuts from the anterior to posterior positions along the sagittal osteotomy. (**B**) The posterior mandibular border is split along the mylohyoid groove. (**C**) The buccal side of the mandible is split. (**D**) The split is completed with a wide osteotome. (**E**) The proximal segment is retracted laterally to expose the surgical site. (**F**) Marked holes are connected with a Lindeman bur. (**G**) The ostectomy is completed with a reciprocating saw. (**H**) The mandibular angle ostectomy is completed.

Horizontal, sagittal, and vertical osteotomies were performed in accordance with the modifications by Hunsuck^7^ and Epker^8^. An additional osteotomy was conducted vertical to the horizontal osteotomy (Fig. 2). The split procedure was modified. A kocher forcep was placed over the anterior border of the mandibular ramus to retract the soft tissue superiorly. First, anterior-to-posterior osteotomy cuts along the sagittal osteotomy were defined with a straight chisel, thus ensuring that the osteotomy was completed through the cortex and down to the cancellous bone (Fig. 3A). Next, a slightly curved chisel was positioned to straddle the horizontal osteotomy. Then, force was applied to the chisel with a bone mallet. The proximal and distal segments were separated initially at the posterior of the foramen (Fig. 3B). Thereafter, the slightly curved chisel was placed in the anterior part of the sagittal cut. Force was applied perpendicular to the inferior mandibular border. The proximal and distal segments were then separated at the buccal side (Fig. 3C). Finally, the split was completed with a wide osteotome rotated clockwise (Fig. 3D).

**Figure 4:**
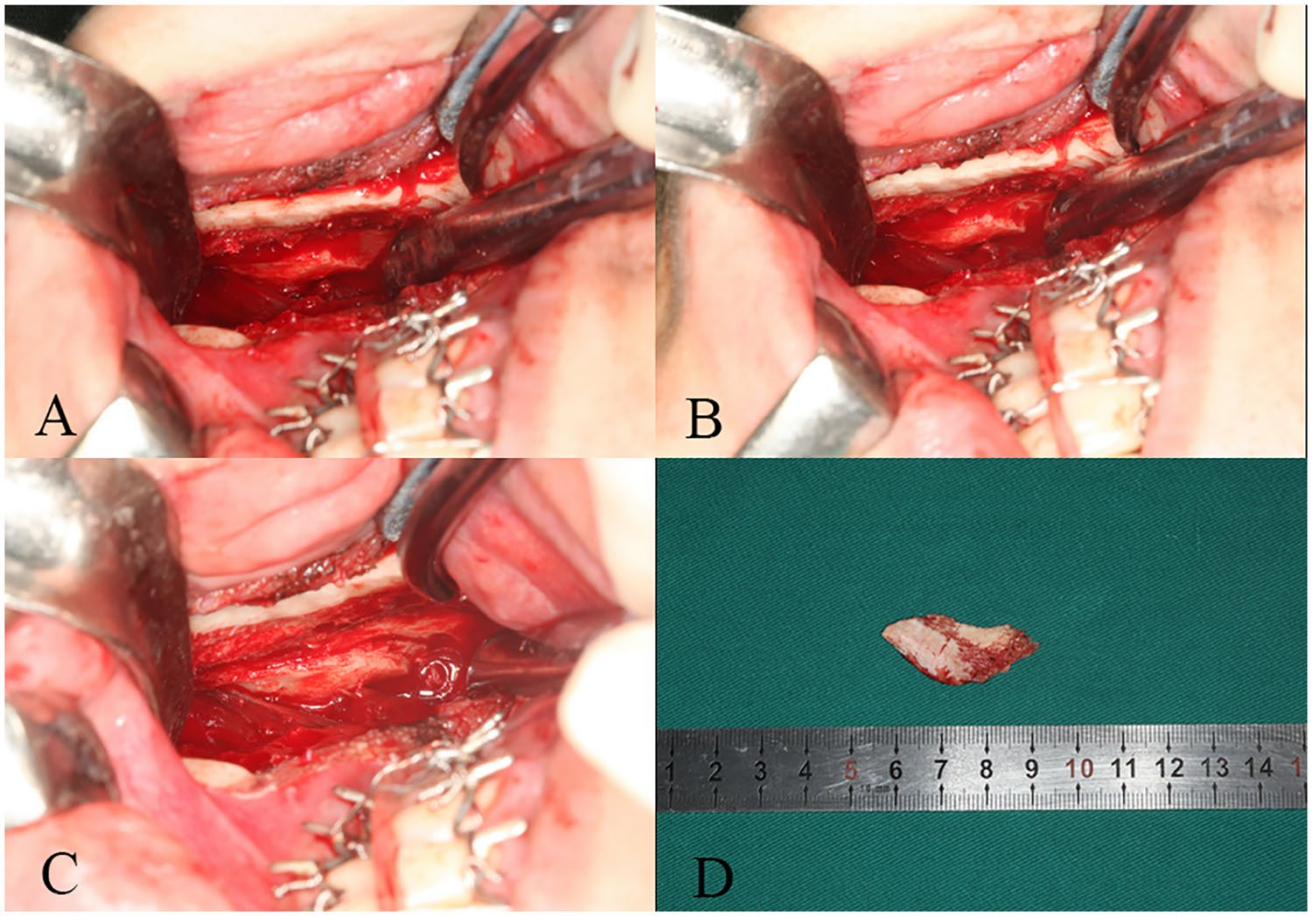
Clinical photographs of mandibular angle ostectomy. (**A**) The mandibular angle is left intact with the proximal segment. (**B**) The osteotomy line is marked with a Lindeman bur. (**C**) The ostectomy is completed with a reciprocating saw. (**D**) The mandibular angle is removed from the medial side of the mandible.

The proximal segment was laterally retracted to enlarge the gap between segments (Fig. 3E). The medial pterygoid muscle and the stylomandibular ligament were stripped from the medial side of the proximal segment. The osteotomy line was marked with a round bur in accordance with the preoperative design. The line started from the posterior margin of mandibular ramus on the occlusal plane and proceeded inferiorly and anteriorly to the antegonial notch. Drill holes were connected with a Lindeman bur only at the medial side of the cortex (Fig. 3F). The ostectomy was completed with a reciprocating saw through the bicortical bone of the mandibular angle (Fig. 3G). The angle was then removed through the medial side of the mandible (Fig. 3H). Finally, any remaining sharp edges were trimmed with a round bur. Fixation was not needed in this area. A clinical photograph of the mandibular angle ostectomy is shown as Fig. 4. After the procedure, sustained-suction drainage was retained in the bilateral mandible for 24 h.

### Data collection

Standardized lateral cephalometric radiographs were routinely obtained for all the patients at the following four stages: (1) within 1 week preoperatively (T0); (2) 3–5 days postoperatively when the desired occlusion was obtained (T1) to assess surgery-related changes; (3) 3 months postoperatively (T2) to assess short-term adaptive changes; (4) at the latest follow-up (10 months on average, T3), to assess long-term adaptive changes.

**Figure 5:**
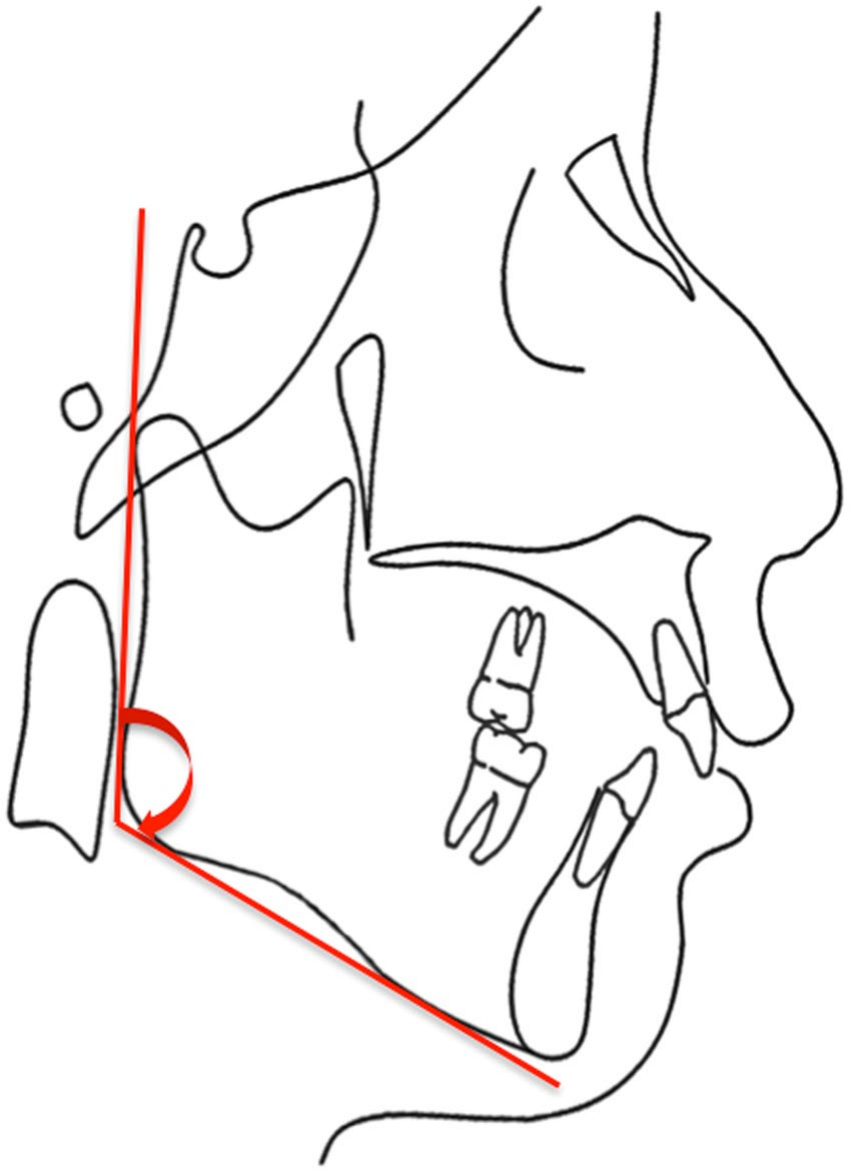
Tangential lines to the posterior and inferior borders of the mandible. The angle between these two lines was defined as the mandibular angle.

Tangential lines to the posterior and inferior borders of the mandible were drawn, and the angle between the two lines was defined as the mandibular angle (Fig. 5). The mandibular angle was measured thrice, and the mean value of measurements was used for statistical analysis.

### Statistical analysis

Data were statistically analyzed using SPSS (version 20.0 for window). The same investigator repeated measurements at least 2 weeks apart to assess the reliability of the method. Paired t-test was used to assess systematic error, and the Dahlberg formula^15^ was used to calculate random error.

Mandibular angle measurements at T0, T1, T2 and T3 were compared by repeated-measures ANOVA (p = 0.05). Pair-wise multiple comparisons were conducted through Bonferroni correction (p = 0.05).

### Data Availability

All data generated or analysed during this study are included in this published article.

### Informed consent statement

Informed consent was obtained from each subject. The study was approved by the Institutional Review Board of Peking University School and Hospital of Stomatology.
